# Multi‐system resilience for disabled children and their families during crisis and emergency

**DOI:** 10.1111/dmcn.16158

**Published:** 2025-01-13

**Authors:** Tali‐Noy Hindi

**Affiliations:** ^1^ Political Sciences University of Haifa Haifa Israel

## Abstract

This commentary is on the original article by Merrick et al. on pages 676–687 of this issue.

Emergency management is complex, requiring different layers of actions taken by diverse actors. Emergency activities such as evacuations, assistance, and casualty management issues are undertaken by government agencies, civil society organizations, and private‐sector companies that can be used to finance, build, operate projects, and provide emergency services. Merrick et al.^1^ have established agreement on specific policies and practices for services supporting disabled children during emergencies. The authors revised these solutions into draft recommendations focused on localized decision‐making in three key areas: (1) prioritizing care for high‐risk health issues; (2) organizing and delivering care; and (3) communicating changes in care provision during future emergencies. This article is fundamental since it underscores the fact that acute crises no longer have a geographically localized impact; their influence expands to far‐reaching locations due to globalization. Hence, it is vital to prepare for acute crises that will almost certainly occur in the future.

Moreover, different governmental ministries, regional and local authorities, and service providers are responsible for preparing services, to operate in routine and emergency situations, and enhancing their resilience. The recommendations in the Merrick et al. study include cross‐service and sector provision such as the ‘Every Contact Counts’ approach. However, research shows that devolution of power to local governments in crisis is only partially effective. During an ongoing acute crisis, budget cuts and reallocation should be expected, thus forcing local authorities to reduce services provided routinely to residents. This reduction would inevitably be even higher in weaker local authorities.^2^

Most recommendations emphasize the prevalent approach in rehabilitation psychology studies that describes the effects of crisis and distress on the individual. However, a disabled child's low level of functioning has far‐reaching consequences for family members, just as a low level of family functioning affects the health and well‐being of the disabled child. Therefore, current policy studies refer to family resilience as the reactivity of the family to complex challenges such as the effects of climate change, epidemics, or war.^3^

The impact of crises on families with disabled children is multidimensional. Families face many challenges that are exacerbated by the complex circumstances of caring for disabled children. Intersecting factors that endanger or promote family resilience describe how marginalized groups face multiple layers of risk that interact with low‐resource intensity.^4^

The concept of coping with multiple stressors arising from disruptions and threats across various systems is referred to as multisystemic resilience, where the resilience of one system can influence another.^5^ For example, a disabled infant who has complex physical and intellectual disabilities or a rare syndrome attends an early intervention daycare center. The infant and their family receive different services at the daycare, such as therapeutic services, developmental services, and emotional therapy. During a pandemic, rehabilitative daycare can be shut down, affecting three central systems: education, healthcare, and welfare. In this situation, families with disabled children have to look for service providers to assist the children in their homes. However, research on integrative multi‐system resilience remains limited. Future studies should evaluate how to synchronize different systems during a crisis, understand the interactions between systems, and assess their combined impact on family resilience. A key challenge is identifying profiles of multi‐risk families and developing tailored policy packages for each. This approach necessitates proactive involvement from government ministries in supporting families with multi‐factorial risk.

## Data Availability

Not required.
